# He for she? Variation and exaggeration in men's support for women's empowerment in northern Tanzania

**DOI:** 10.1017/ehs.2021.23

**Published:** 2021-03-18

**Authors:** David W. Lawson, Susan B. Schaffnit, Joseph A. Kilgallen, Yusufu Kumogola, Anthony Galura, Mark Urassa

**Affiliations:** 1 Department of Anthropology, University of California, Santa Barbara, California, USA; 2 National Institute for Medical Research, Mwanza, Tanzania

**Keywords:** Gender equality, patriarchy, sexual conflict, social norms, global health, marriage

## Abstract

Achieving gender equality fundamentally requires a transfer of power from men to women. Yet data on men's support for women's empowerment (WE) remains scant and limited by reliance on self-report methodologies. Here, we examine men's support for WE as a sexual conflict trait, both via direct surveys (*n* = 590) and indirectly by asking men's wives (*n* = 317) to speculate on their husband's views. Data come from a semi-urban community in Mwanza, Tanzania. Consistent with reduced resource competition and increased exposure to relatively egalitarian gender norms, higher socioeconomic status predicted greater support for WE. However, potential demographic indicators of sexual conflict (high fertility, polygyny, large spousal age gap) were largely unrelated to men's support for WE. Contrasting self- and wife-reported measures suggests that men frequently exaggerate their support for women in self-reported attitudes. Discrepancies were especially pronounced among men claiming the highest support for WE, but smallest among men who held a professional occupation and whose wife participated in wage labour, indicating that these factors predict genuine support for WE. We discuss the implications of these results for our understanding of both individual variation and patriarchal gender norms, emphasising the benefits of greater exchange between the evolutionary human sciences and global health research on these themes.

## Graphical abstract

**Figure:**
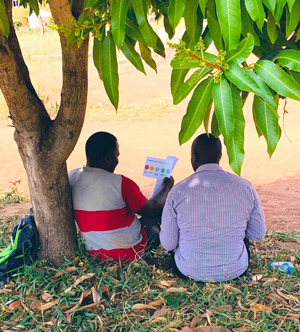

**Social media summary:** Husbands and wives disagree when evaluating men's support for women's empowerment, indicating frequent exaggeration of support by men

## Introduction

Women's empowerment (WE), defined here broadly as the advancement of women's autonomy, rights and wellbeing, is a central goal of contemporary global health. In recent years, attention has turned to the idea that men, often presumed to benefit only from defending the status quo of patriarchal regimes, may be effectively enrolled as agents of change (Barker et al., 2010; Cihangir et al., 2014; Connell, 2005; Estevan-Reina et al., 2020; Flood, 2015; Subašić et al., 2018; Sudkämper et al., 2020; Vaillant et al., 2020). The United Nations’ ‘*HeforShe*’ campaign, for example, encourages men to publicly declare solidarity with women in the fight for gender equality (UN Women, 2014). However, our understanding of what makes some men more supportive of women than others remains limited, particularly among relatively low-income nations (Charles, 2019; Levtov et al., 2014). Addressing this gap, we contribute novel data on male attitudes to WE in a semi-urban community in northwestern Tanzania.

We test several hypotheses about the sociodemographic determinants of men's support for women. We also tackle an important methodological limitation of existing research, namely the role of social desirability bias in the (mis)measurement of men's attitudes (i.e. the tendency of survey respondents to answer questions in a manner that will be viewed favorably by others). While we draw on studies from across the social sciences, we frame our research around the notion of WE as a sexual conflict trait, aiming to promote greater connections between evolutionary human science and global health scholarship on gender norms and ideology. Anthropologists and economists in particular have long considered the socioecological and evolutionary roots of patriarchy, often placing emphasis on the role of livelihood shifts, such as the uptake of agriculture and its impacts on gendered divisions of labour and resource control, along with variation in post-marital residence norms which may influence a woman’s ability to draw on support from kin (e.g. Alesina et al., 2013; Becker, 2019; Boserup, 1970; Draper, 1975; Hansen et al., 2015; Hrdy, 1997; Smuts, 1995). However, research in this tradition has rarely considered individual variability in men's support for WE *within communities*.

If WE is best understood as a sexual conflict trait (i.e. bad for men, good for women), then a simple prediction would be that all men will oppose WE. This is naive on several fronts. First, inclusive fitness interests are spread among kin of each sex (Brooks & Blake, 2019). In other words, a man must consider not only the consequences of WE for himself, but also those for his mother, sisters and daughters (and likewise a woman's attitudes will be influenced by considering their father, brothers and sons). As such, we can anticipate that men and women's attitudes will often converge, and potentially be sensitive to individual variation in the gender composition of kin, of which there is some limited evidence (e.g. Borrell-porta et al., 2019; Brooks & Blake, 2019; Oswald & Powdthavee, 2010). Second, men's fitness requires effective cooperation with women in reproduction and rearing offspring, typically involving coordinated production and consumption of shared resources. As such, some aspects of WE may be beneficial to men. For example, there is evidence that women's social status positively predicts child health (Alami et al., 2020). More generally, if a particular domain of WE does not dictate that resources are allocated away from husbands specifically or men more generally, a conflict of interests may be absent. Finally, individual attitudes and actions are fundamentally influenced by cultural transmission of beliefs (Creanza et al., 2017; Mesoudi, 2011). A solid understanding of men's support for WE therefore must consider the transmission of gender ideology, which may be adopted as a group-level norms shared by both genders, rather than be differentiated by women and men within society.

To date, global health scholarship on men's support for WE in low-income nations has drawn primarily on three sources of data. It is useful to take stock of these studies, before laying out the theoretical orientation of our hypotheses. First, several studies consider attitudes to intimate partner violence (IPV) specifically, as measured in the Demographic and Health Surveys and related cross-national databases (e.g. Lawoko, 2008; Sardinha & Catalán, 2018; Tran et al., 2016; Uthman et al., 2009). Men living in relatively urban areas, wealthier and more educated men are generally less likely to openly declare IPV to be acceptable (Tran et al., 2016; Uthman et al., 2009). However, the magnitude and direction of relationships vary, e.g. one study reports that higher educational attainment is associated with greater approval of IPV in Zambia (Lawoko, 2008).

National attitudinal surveys make up a second key source of data (Charles, 2019; Kyoore & Sulemana, 2019; McDaniel, 2008; Seguino, 2007). Kyoore and Sulemana (2019), for example, using the *World Values Survey*, report that highly educated individuals are more supportive of gender equality in the domains of education, employment and politics across five African countries, but did not stratify their analysis by gender. Charles (2019), using data from the *Afrobarometer* surveys, considered men's agreement with the statement ‘In our country, women should have equal rights and receive the same treatment as men do’ as a measure of men's unqualified endorsement for equal opportunity egalitarianism across 34 African nations. Overall, relatively well-educated men were more likely to support this indicator of gender equality, as were men who lived in urban areas, or had internet or phone access. However, there was no relationship between support for gender equality and subjective social class (measured as perceived material advantage).

Finally, more comprehensive surveys with a dedicated focus on documenting men's attitudes to WE across multiple domains have recently emerged. Chief among these is the *International Men and Gender Equality Survey* (IMAGES), now carried out across multiple, primarily low- and middle-income countries, including Tanzania (Levtov et al., 2018). Compiling IMAGES data from over 10,000 men from Brazil, Chile, Mexico, India, Bosnia and Herzegovina, Croatia, Democratic Republic of Congo, and Rwanda, Levtov et al. (2014) examined a composite indicator summarising attitudes to gender roles, IPV, sexuality and reproductive health. Greater education attainment predicted relatively more equitable attitudes in all eight countries. However, relationships between men's income and support for WE were mixed (positive in four nations, negative in a fifth and absent in three nations). Estimated effects of age, marital status and employment were also mixed and mostly statistically non-significant. As such, an understanding of the correlates of men's support for WE remains elusive. Nevertheless, Levtov et al. (2014) demonstrate that men who report gender-equitable attitudes to WE also report gender-equitable practices, including greater participation in domestic duties, and reduced IPV. This suggests that men's attitudes meaningfully correspond with behaviour, justifying further investigation (see also Fleming et al., 2015). However, this conclusion, and the findings reviewed so far, are undermining by the possibility that social desirability bias may lead men to misrepresent their attitudes and/or behaviour in self-report surveys.

Here, we present findings from a survey of men's attitudes in a semi-urban community in northern Tanzania. While focus on a single community sample limits generalisability, it also has important advantages. Most notably, we avoid aggregating data across heterogeneous subpopulations, typical of the analyses described above. Instead we draw inferences about men's support for WE by comparing literal neighbours, limiting scope for statistical confounding at individual and ecological levels. We can also embed interpretation of our findings in a solid understanding of this specific cultural context, based on our prior research in this setting (see Methods). Furthermore, our study is distinguished by (a) considering a broad range of sociodemographic variables and (b) explicitly addressing social desirability bias, through the use of a simple, but novel *indirect* methodology: asking men's wives to speculate on their husband's attitudes to WE.

### Hypotheses

With respect to socioeconomic variability, we consider men's education, wealth, occupation and his wife's earnings. Prior scholarship has argued that schooling in low-income settings increases exposure to, and adoption of, equitable gender norms (Charles, 2019; Kyoore & Sulemana, 2019; Levtov et al., 2014). We find this logic appropriate for this setting where both strong patriarchal norms are evident, and education, along with various forms of modern media, probably increases exposure to relatively egalitarian ‘global cultural scripts’ (Pierotti, 2013). However, we caution against simplistic notions of a linear progression towards greater support for WE accompanying ‘modernisation’. For example, it has long been recognised that foraging is associated with relatively egalitarian gender norms in contrast to more recently acquired subsistence modes (Draper, 1975), and there is considerable diversity in control over women's sexuality across small-scale societies (Scelza, 2013). Beyond shifting norm exposure, greater material wealth is anticipated to relax gender-based conflict over resources shared between spouses, thereby increasing men's support of WE (Levtov et al., 2014). Combining elements of both mechanisms, we furthermore anticipate that men reliant on subsistence farming will be less supportive of WE than men who hold relatively high-status professions that bring both greater wealth and greater exposure to extra-local cultural norms. Finally, women's employment is expected to directly influence a wife's bargaining power, as she now contributes more capital to the household budget, which may consequently lead husbands of women who earn wages to offer relatively greater support for WE (Seguino, 2007).

With respect to demographic variability, we consider age, fertility, marriage type (monogamous or polygynous) and spousal age gap. Brooks and Blake (2019) predict that older men will be more likely to adopt sociopolitical positions which support women because their inclusive fitness becomes less tied to their own direct fitness and more dispersed across kin of both genders as they age. Similarly, it has been argued that larger family size could increase a man's support for WE via increasing the specific likelihood that he has daughters (Charles, 2019). Note, we do not explore how individual variation in gender composition of family members relates to men's attitudes in this manuscript. This is the subject of separate dedicated analysis (in progress) designed to explore alternative ideas about the impact of kin gender on support for WE. In contrast, high fertility is anticipated to reinforce a strong division of labour between the sexes, since the demands of pregnancy, lactation and childcare fall primarily on women, potentially lowering men's support for WE. Prior studies have linked multiple measures of low WE with high fertility (e.g. Upadhyay et al., 2014), but less is known about relationships with men's attitudes. Higher fertility may also be indicative of men exerting direct coercion over reproduction, assuming that women would otherwise prefer lower fertility than men owing to the, potentially life-threatening, substantial physical costs of pregnancy and childbirth (Stieglitz et al., 2018, but see Moya et al., 2016).

In global health research, both polygyny and large spousal age gaps are widely assumed to occur at a detriment to women and contribute to gendered-power inequalities (Barbieri et al., 2005; Lawson & Gibson, 2018). This leads to the prediction that men in such marriages will be relatively less supportive of WE. Yet evolutionary human scientists have highlighted mixed associations between these practices and wellbeing, along with context-dependent scope for either male coercion and/or female choice in both polygyny (Borgerhoff Mulder, 1992; Lawson et al., 2015; Strassmann, 1997; Uggla et al., 2018) and in marriages to older men (Conroy-Beam & Buss, 2019; Lawson et al., 2021). In particular, where such marriages occur without coercion and enable women to partner with relatively wealthy men, they may be advantageous or neutral for women, at least within the scope of limited available options, rather than necessarily being at odds with WE. In recognition that polygyny and large spousal age gap are best considered *potential* rather than definitive indicators of sexual conflict, our analysis of the relationship of these measures to WE is best considered exploratory.

Social desirability bias is a widely acknowledged limitation of existing scholarship on men's support for WE (Charles, 2019; Lawoko, 2008; Levtov et al., 2014; Schuler et al., 2011; Vaillant et al., 2020), and evidenced by common results such being interviewed by a woman rather than a man increasing the likelihood of endorsing gender equality (e.g. Charles, 2019). It may also make sense of some surprising findings. For example, women frequently self-report greater acceptance of IPV than men, particularly in regions where IPV is most prevalent (Sardinha & Catalán, 2018; Tran et al., 2016; Uthman et al., 2009). This is consistent with women internalising harmful cultural norms, but is also indicative that men may downplay their true attitudes to avoid being viewed negatively by interviewers. It is less well appreciated that social desirability bias is especially problematic because if some men misrepresent their beliefs *more than others*, the validity of established sociodemographic comparisons is undermined. Where innovative methodologies have addressed this issue, cause for concern is confirmed. For example, Gibson et al. (2018), used an unmatched count technique in rural Ethiopia, whereby participants selected the number of items they approved of from a list without specifying support for individual items. Contrasting participant responses based on whether or not this list includes particular items then enables hidden preferences to be measured without sacrificing participant anonymity. Using this method, Gibson et al. concluded that men (and women) often misrepresent their true attitudes to female genital cutting (FGC). Relatively educated men, presumably most aware of the desirable answer, were most likely to exaggerate opposition to FGC under direct questioning.

We use a novel approach to this issue combining self-reported attitudes with an indirect wife-reported measure, whereby wives speculate on their husbands’ attitudes. The discrepancy between measures is then used to estimate the extent to which men misrepresent their true attitudes. This assumes that wife-reported measures are accurate, or at least more accurate, than men's self-reported attitudes, a not unreasonable assumption we return to in our discussion. It also enables discrepancies between self-report and indirect measures to be identified at the individual level, while the unmatched count technique can only do so at aggregate levels, limiting analytical scope (Gibson et al., 2018). Following Gibson et al., we hypothesise that higher socioeconomic status men will be most likely to exaggerate support for women, and that exaggerated support will be most pronounced in interviews conducted in the presence of a foreign researcher (i.e. the US-based co-authors of this study), assuming that participants will be most comfortable reporting true attitudes to researchers of the same nationality.

## Methods

### Context

All data were collected within a single semi-urban community within the boundaries of the Magu Health and Demographic Surveillance System (HDSS), situated in northwestern Tanzania, approximately 20 km east of Mwanza city. The HDSS has monitored the population of over 35,000 residents since 1994 (Kishamawe et al., 2015). The overwhelming majority of residents identify as Christian and Sukuma (Hedges et al., 2018), an ethnic group representing approximately 17% of Tanzania (Malipula, 2014). Traditionally, the Sukuma relied on subsistence agropastoralism. Today, petty trade in agricultural products remains the predominant source of income, but consumer goods have become an important indicator of wealth (Wijsen & Tanner, 2002). Following urbanisation and labour market diversification, the largest settlement, Kisesa, from which all data in this study was collected, is now best described as a town (Hedges et al., 2018).

Marriages vary in formality, but almost always involve cohabitation (Schaffnit et al., 2019b; Wight et al., 2006). Formal marriages typically include bridewealth, with larger transfers for younger brides (Schaffnit et al., 2019a). Polygyny is permitted and most common in relatively rural areas (Lawson et al., 2021). Virginity is not a pre-requisite for marriage, and childbearing before or outside of marriage is common (Boerma et al., 2002), as is transactional sex (Wamoyi et al., 2019). A double standard applies to sexual behaviour; a woman's reputation may be damaged if she is seen as promiscuous, while men's reputation is generally enhanced by greater sexual activity. Extra-marital affairs are typically blamed on women not men (Wight et al., 2006). IPV is common; around 2/5 of women participating in this study self-reported experience of IPV within the last year. Women report autonomy in their choice of marriage partner, albeit within the context of constrained options that limit viable roles for unmarried women (Schaffnit et al., 2019b). Marriage frequently occurs during adolescence and spousal age gaps can be large (Lawson et al., 2021; Schaffnit et al., 2019a, b), potentially reinforcing gendered power inequalities. Divorce may be initiated by either partner, and is typically followed quickly by remarriage, at least for women of childbearing age (Boerma et al., 2002). Notions of male authority and women's subordination in marriage are reinforced and reflected in traditional Sukuma songs (Masele & Lakshmanan, 2021)

Sukuma families traditionally follow patrilineal inheritance and patrilocal post-marital residence, but norms are flexible (Wijsen & Tanner, 2002), and influenced by recent urbanisation (informal observations indicate an increase in neolocal residence). Girls’ education has increased substantially in recent cohorts, now matching or exceeding boys’ education within some communities (Hedges et al., 2018). While schooling reduces farm and household work, this is less true for girls, who even while attending school contribute substantially to domestic chores (Hedges et al., 2018). Self-report data on direct care of children indicates slightly preferential treatment of sons by fathers, but not mothers (Hassan et al., 2019). Fostering, especially among close kin, is common; around a quarter of children between 7 and 19 years live apart from both parents, and girls are more often fostered than boys (Hedges et al., 2019; Urassa et al., 1997).

### Sampling

Data collection occurred between June and August 2019. The 2018 HDSS was used as a sampling frame, identifying resident married menaged 25–40 years who had at least one living child. A limited age range was chosen to maximise our ability to explore variation in attitudes independently of potential age/cohort effects. Married men with children only were selected to allow for comparisons between self-reported and wife-reported measures, and to enable additional analyses of the relationship between the child gender and support for WE (the subject of a separate paper). These selection criteria are unlikely to meaningfully bias our sample given local norms of early marriage and childbearing. We identified 1,275 eligible husband–wife pairs and initially aimed to randomly sample 1,000 men and 400 wives, reflecting our primary focus on men and budgetary constraints. Sampling men, however, even with HDSS identifying information, proved challenging given frequent daily movements and longer-term migrations. We therefore settled on a convenience sampling approach, attempting to sample all eligible and available men, ultimately achieving a final sample of 590 men and 317 of their wives.

We visited each man's residence and, if he was present, interviewed him on location. If absent, present family or neighbouring community members were asked about his whereabouts (typically his place of work), and if within the immediate range of Kisesa, the man was visited. Wives were more commonly found at home, but were also tracked if absent. In rare cases of polygyny, one wife was chosen for interview, based on immediate availability. Among initially targeted but unsampled men (*n* = 685), 61% were not included because we could not easily establish their current location. In other cases, the man was excluded because were informed he had moved since the last HDSS (18%), had temporarily travelled away from Kisesa (13%, e.g. to visit relatives), he was found to be ineligible (4%, e.g. had recently divorced or had died) or he refused to participate (4%). Occasionally, men provided ages that did not match the HDSS. If the man's self-reported age at the time of survey was within 5 years of our selection criteria, he was included in our final sample (i.e. men aged 20–45 years are included). Sampled men were on average 0.8 years older than men who were eligible but not sampled (*t*(1251) = −3.48, *p* < 0.001). Thus, our sample is biased to only slightly older men, as well as men who work at or near their home.

### Surveys

Surveys were completed on tablets using Open Data Kit (Hartung et al., 2010). Participants provided informed consent and were interviewed in private by Tanzanian researchers of the same gender, in Swahili. Men were asked to self-report sociodemographic characteristics, including cash income, but relative wealth was assessed by research staff, all of whom were very familiar with the Kisesa area, based primarily on the men's quality of housing and attire. This estimate, ranging from very wealthy to very poor, is subjective, but simple to employ and not subject to the same biases as asking participants to self-rate wealth. We lack a direct validation of this measure, but in our experience community members prefer not to be viewed as either unusually wealthy or poor, undermining self-reported comparisons. Asking poor households to compare themselves with wealthier neighbours also undermines the dignity of respondents, especially given that the field team are also viewed as exceptionally wealthy.

**Figure 1.**
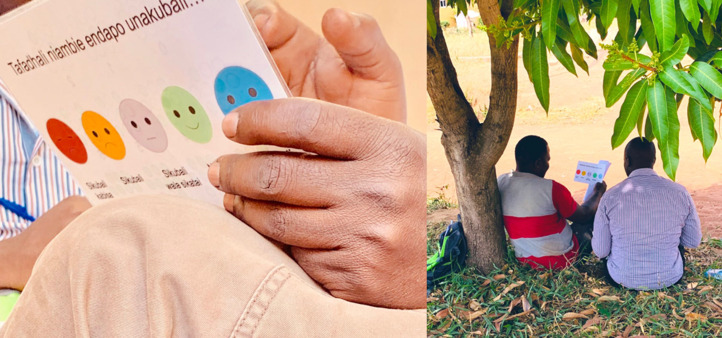
A participant survey. Men were asked to report their relative agreement or disagreement with 20 questions relating to women's empowerment (see Figure 2). A visual aid of possible responses (*strongly agree, agree, neither agree nor disagree, disagree, strongly disagree*) was used to reinforce the use of all available response options.

To assess support for WE men were read 20 statements (see Results for details) and asked to state if they *strongly agree*, *agree*, *neither agree or disagree, disagree* or *strongly disagree* with each (Figure 1). When a participant stated that they agreed/disagreed they were asked to clarify whether this was a mild or strong level of agreement/disagreement. The 20 statements were initially selected from components of the ‘Women's Empowerment – Multidimensional Evaluation of Agency, Social Capital & Relations’ instrument (CARE USA, 2014), and then further adapted to suit this context (e.g. we included an additional statement on a woman's ability to prevent her husband taking a second wife). Statements were chosen to span the topics of authority in decision-making, IPV, responsibility in childcare and family planning, women's economic independence, involvement in community affairs, sex-biases in parental care and the viability of women's roles beyond marriage and motherhood (see Supporting Information Table S1 for Swahili translations). Sampled wives were then asked to report, in private and by a same-gender Tanzanian researcher, if they felt their husband would agree/disagree with each statement.

### Analysis

All analyses were implemented in R (R Core Team, 2020). We first describe the characteristics of our sample. We then plot responses to each statement from the attitudinal surveys (using the ‘Likert’ package) and use Wilcoxon rank-sum tests to contrast men's self-reported and wife-reported responses. Although our primary focus is on overall levels of support for WE, we make qualitative comparisons regarding the degree of agreement across statements and domains of WE, interpreted in light of the phrasing of each statement. We also include a supplementary analysis that quantifies discrepancies between self-reported and wife-reported attitudinal survey responses for each statement (i.e. means and standard deviations). To do this we score individuals 1–5 for all statements from strongly disagree to strongly agree, respectively, where greater agreement is more supportive of WE, and reversed code scores where the opposite is true, and then subtract the self-report score from the wife-support score. While useful in identifying the source of overall discrepancies in support for WE between self- and wife-reported measures, we note that, owing to differences in the phrasing of statements (and so precision in measuring latent constructs of interest), the comparative magnitude of such discrepancies should be interpreted with caution.

Internal consistency across responses to each attitudinal statement included in our survey is assessed using Cronbach's *α*. We calculated a summary score for both the self-reported and wife-reported responses. As above, we coded individual items ranging from strongly disagree to strongly agree as 1–5, respectively, for all statements where greater agreement is more supportive of WE, and reverse coded where the opposite was true. Individual scores are then summed so that a score of 100 indicates the strongest possible support. For those who responded ‘don't know’ or refused to answer up to five statements, a multiplication factor was applied to the sum total so that the maximum possible score equals 100, while participants with five or more ‘don't know’ or ‘refuse’ responses are excluded from analysis. A *discrepancy score*, measuring the difference between the summary self-report and wife-reported scores, was computed by subtracting the former from the latter; a larger score is indicative of relatively exaggerated support for WE in men's self-reported attitudes compared with their wife's estimate.

Associations between men's characteristics and each summary score are assessed via simple and multivariate linear regression. All independent variables are included as categorical predictors to enable the exploration of potential threshold effects. First, we examine bivariate associations and summarise results visually using forest plots. To establish whether bivariate relationships are additive, all independent variables which demonstrated significant bivariate associations with either summary score are carried forward to multivariate linear regression. Two models predicting men's self-reported summary score were run: model one excludes and model two includes wife's income because this data is only available for the subsample of men whose wives were sampled. A third model then explores relationships with our independent variables with the wife-reported summary score, and a final model predicts the discrepancy score as the outcome to assess differences in the self- and wife-reported summary scores (hypothesised to primarily indicate men's misrepresentation of support for WE).

### Ethical approval

Ethical approval for this study was granted by UCSB's Office of Research (4-19-0247), the Tanzanian National Institute for Medical Research Lake Zone Institutional Review Board (MR/53/100/595) and the Tanzanian National Ethical Review Committee (NIMR/HQ/R.8a/Vol.IX/3104).

## Results

### Men's characteristics and support for women's empowerment by item

**Figure 2.**
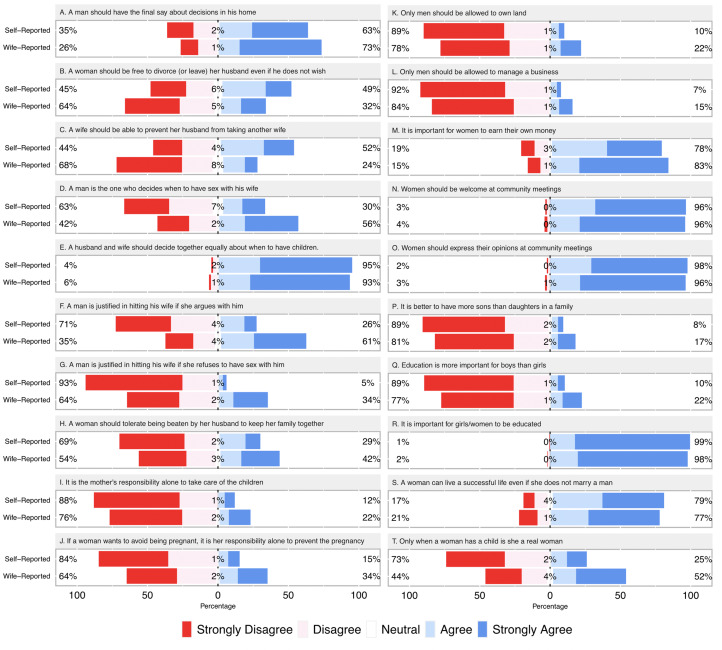
Self and wife-reported views on women's empowerment. Figure shows sum percentage which *strongly disagreed* or *disagreed* on left side, and sum percentage which *agreed* or *strongly agreed* on right side for each statement. Maximum sample sizes are 590 for self-reported and 317 for wife-reported responses. See Supporting Information Table S3 for complete descriptive data and Wilcoxon rank-sum tests for statistical differences between self-report and wife-reported measures (*p*-values reported in the main text).

Table 1 provides descriptive statistics on sampled men (*n* = 590). Men's characteristics did not differ between those that were sampled alone and for those whose wife also provided data, with the exception that the latter were more likely to report skilled labour occupations and slightly higher fertility (Supporting Information, Table S2). Figure 2 displays men's self-reported response to each statement alongside wife-reported measures. Self-report responses from the full sample and the subsample of men for whom paired wife-reported data is available had near identical distributions (Supporting Information Figure S1). We therefore utilise data from the full sample of men in these contrasts and throughout our analyses. Full descriptive data and Wilcoxon rank-sum tests comparing the distribution of self- and wife-reported responses are provided in Supporting Information Table S3 (for the sake of brevity, corresponding *p*-values only for these contrasts are reported here in the main text). In a small proportion of cases participants refused to answer specific statements or responded that they did not know about their own or husband's beliefs (0–1% of self-report and 4–12% of wife-report cases). These cases are excluded from presentation and further analysis of individual attitude questions.

**Table 1.** Characteristics of sampled men (*n* = 590)

**Variable**		***n* (%)**
**Age** (years)	25–30	103 (17.5%)
	30–34	174 (29.5%)
	35–39	209 (35.4%)
	40–45	104 (17.6%)
**Education** (highest level achieved)	No education	17 (2.9%)
	Primary school	397 (67.4%)
	Secondary school	133 (22.6%)
	Technical training	9 (1.5%)
	Higher education	33 (5.6%)
**Occupation**	Subsistence	61 (10.6%)
	Unskilled labour	118 (20.5%)
	Skilled labour	358 (62.2%)
	Professional	39 (6.8%)
**Income** (Tanzanian Shillings in past week)	None	99 (16.9%)
	Low (<30,000)	149 (25.4%)
	Medium (30,000–59,999)	175 (29.9%)
	High (>60,000)	163 (27.8%)
**Wife's income** (past week)	Wife has no income	192 (60.8%)
	Wife earns income	124 (39.2%)
**Subjective wealth rating**	Very poor	1 (0.2%)
	Poor	177 (30.0%)
	Average	323 (54.7%)
	Wealthy	74 (12.5%)
	Very wealthy	15 (2.5%)
**Number of children**	1–2	176 (29.8%)
	3	137 (23.2%)
	4	110 (18.6%)
	5+	167 (28.3%)
**Spousal age gap**	Wife same age or older	39 (12.3%)
	Wife 1–4 years younger	94 (29.7%)
	Wife 5–8 years younger	118 (37.2%)
	Wife 9+ years younger	66 (20.8%)
**Marriage Type**	Monogamous	554 (93.9%)
	Polygynous	36 (6.1%)
**Mzungu present?**	No	443 (75.1%)
	Yes	147 (24.9%)
Data is missing for 1/590 case for Education, 14/590 cases for Occupation, 4/590 cases for Income, and 43/317 cases for Wife's income. All other variables have complete data.		

Questions on decision-making (statements A–E, Figure 2) reveal widespread belief in male authority. A substantial proportion of men stated that they should have final say about decisions in their home (self-report, 63%; wife-report, 73%; *p* < 0.001), that women should not be free to divorce without a man's consent (self-report, 45%; wife-report, 64%; *p* < 0.001) or be able to prevent a man from taking another wife (self-report, 44%; wife report, 68%; *p* < 0.001), and that the man should be the one to decide when to have sex (self-report, 30%; wife-report, 56%; *p* < 0.001). Across these statements, self-report responses indicated significantly greater support for WE than wife-reported measures. Only when asked about having a child did the overwhelming majority of men believe that this is a decision that should be made equally by a husband and wife (self-report, 95%; wife-report, 93%; *p* = 0.18). Intimate partner violence was openly justified as an acceptable means of resolving marital disagreements for many men (statements F–H). This trend held with respect to arguments generally (self-report, 26%; wife-report, 61%; *p* < 0.001) and when a wife refuses sex specifically (self-report, 5%; wife-report, 34%; *p* < 0.001). A sizeable proportion of men (self-report, 29%; wife-report, 42%; *p* < 0.001) also agreed that women should tolerate IPV to keep her family together. For each statement, discrepancies between self- and wife-reported measures are consistent with men exaggerating their disapproval of IPV.

Contrasting the above, men reported greater support for WE in statements reflecting women's responsibilities for childcare and family planning (statements I–J), economic independence (statements K–M) and involvement in community affairs (statements N–O). Relatively few men agreed that women alone should be responsible for childcare (self-report, 12%; wife-report, 22%; *p* < 0.001), or avoiding unwanted pregnancies (self-report, 15%; wife-report, 34%; *p* < 0.001). The large majority of men stated that women should be able to own land (self-report, 89%; wife-report, 78%; *p* < 0.001) and manage a business (self-report, 92%; wife-report, 84%, *p* = 0.13), and agreed that it is important for women to earn their own money (self-report, 78%; wife-report, 83%; *p* < 0.001). The overwhelming majority of men also supported women's attendance at community meetings (self-report, 96%; wife-report, 96%; *p* < 0.001), and felt that women should express their opinions at community meetings (self-report, 98%; wife-report, 96%, *p* = 0.06). For these statements (I–O), self-reported beliefs were again generally more supportive of women than wife-reported measures, although discrepancies are not statistically significant in all cases (Supporting Information Table S3). However, this trend was reversed in one instance (M), with wives actually reporting somewhat higher men's approval of them earning money than men self-reported.

Men had somewhat variable attitudes to parental care (statements P–R) and of women's roles beyond marriage and motherhood (statements S–T). The large majority disagreed with statements that it is better to have more boys in the family that girls (self-report, 89%; wife-report, 81%, *p* = 0.08), that education is more important for boys than girls (self-report, 89%; wife-report, 77%, *p* = 0.001), and that education was important for girls/women (self-report, 99%; wife-report, 98%, *p* = 0.15). Most men agreed also with the statement that women could live a successful life even if unmarried (self-report, 79%; wife-report, 77%, *p* = 0.40). However, men's agreement with the statement that only when a woman has a child is she a real woman was more ambiguous (self-report, 25%; wife-report, 52%; *p* < 0.001), with a clear suggestion that men exaggerate support for WE with respect to this statement.

### Summary scores for self- and wife-reported attitudes

**Figure 3.**
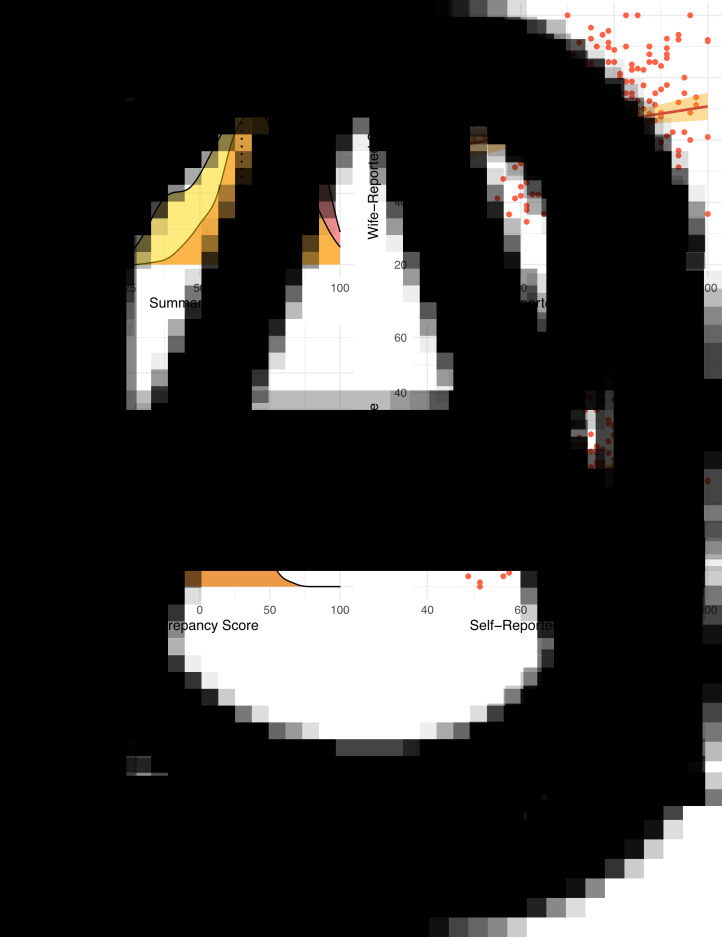
Summary scores for self- and wife-reported support for women's empowerment. **(a)** Density plot for the self- (mean, 75.19; SD, 12.77) and wife-reported scores (mean, 64.97; SD, 16.63). **(b)** Association between the self- and wife-reported scores (Pearson's *r* = 0.16, *p* < 0.01). **(c)** Density plot for the discrepancy score (mean, 10.24; SD, 18.68). Positive scores indicate greater self- compared to wife-reported support for women's empowerment. **(d)** Association between self-reported support for women's empowerment and the discrepancy between self- and wife-reported scores (Pearson's *r* = 0.48, *p* < 0.001).

Attitudinal summary scores could be calculated for pooled individual responses for 589 and 299 self- and wife-reported responses respectively (Figure 3a). Internal consistency across composite questions was good (Cronbach's *α* = 0.77 and 0.82 for self- and wife-reported items respectively). There was no difference in the self-reported summary score between men whose wives were sampled and those whose wives did not provide data (*t* = 0.34, d.f. = 550.87, *p* = 0.73), enabling us to use all cases when contrasting self- and wife-reported scores. Compared with the men's self-reported summary score, the wife-reported summary score indicates lower and more variable support for WE (Welch *t*-test for non-equal variances, *t* = 9.32, d.f. = 481; *p* < 0.001; self-reported mean, 75.19, SD, 12.77; wife-reported mean, 64.97, SD, 16.63). The mean difference (i.e. the discrepancy score) of 10.24 (SD 18.68) can be considered equivalent to a man reporting one level higher agreement (e.g. ‘strongly agree’ rather than ‘agree’) compared with his wife to half (10/20) of all statements.

Self- and wife-reported measures were significantly positively correlated, albeit weakly (Figure 3b, Pearson's *r* = 0.16, *p* < 0.01). This weak correlation is reflected in wide variation in the discrepancy between self- and wife-reported summary scores (Figure 3c). A supplementary analysis (Supporting Information Figure S2) confirms that, while there is a clear overall tendency for wives to report that their husband is less supportive of WE that he self-reports, there is much individual variation in the degree of discrepancy – undermining the overall correlation between summary scores. As might be expected, variability in the discrepancy between self- and wife-reported measures appears greatest for statements in which men held greater viewpoint diversity (e.g. attitudes to decision-making about taking another wife or when to have sex), compared with measures where the large majority of men agree or disagree with a statement (e.g. women's presence at community meetings).

In 71% of cases, the discrepancy score was above or equal to zero, consistent with the notion that men often exaggerate support for WE when self-reporting attitudes. However, in almost a third of cases a wife reported that her husband was *more supportive* of WE than he self-reported, indicating that a discrepancy in scores may also result from alternative mechanisms. The greater the support for WE reported by men, the larger the discrepancy between self- and wife-reported summary scores (Pearson's *r* = 0.48; *p* < 0.001, Figure 3d). This strong correlation implies that men who claim the greatest support for WE exaggerate to a larger degree. However, it also suggests that men who claim exceptionally low support for WE may be exaggerating their *lack of support*, since at this level wives typically reported that their husband is more supportive than he states in self-reported measures.

### Which men are most supportive of women?

**Figure 4.**
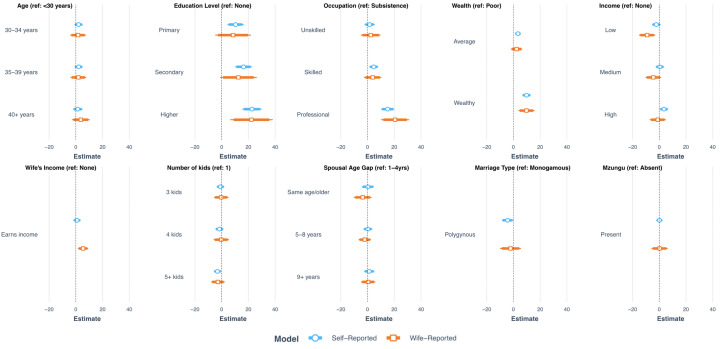
Coefficient estimates from bivariate linear regressions for self- and wife-reported summary scores by men's individual sociodemographic characteristics. Thiner outer bar = 95% confidence intervals; thicker inner bar = 90% confidence intervals.

Neither self- nor wife-reported support for WE was related to a man's age, spousal age gap or the presence of a foreigner (i.e. Mzungu) during the survey in bivariate analysis (Figure 4, Supporting Information Table S4). All other sociodemographic characteristics were significantly associated with at least one summary score. Higher education, a higher-status occupation and greater subjectively rated wealth all predict higher support of WE, across both self- and wife-reported scores. These associations are substantial in magnitude, with for example the difference between subsistence farming and a professional occupation surpassing a standard deviation for both self- and wife-reported scores (*B =* 15.04, 95% confidence intervals (CIs) = 10.12, 19.96; *B* = 20.57, 95% CIs = 10.10, 31.04, respectively, Supporting Information Table S4). Income was related to outcome measures in some contrasts, but the direction was inconsistent. Greater support for WE among men whose wife earned an income was evidenced in wife but not self-reported scores. Men with many children and those in polygynous marriages were less supportive of WE in self-report, but not in wife-reported scores.

Multivariate regression confirms that education, occupation and subjectively rated wealth have largely additive associations with each summary score, although wealth is no longer a significant predictor of wife-reported support for WE in the presence of other socioeconomic factors (Table 2). Note that income was excluded from the multivariate regression because of its inconsistent pattern of association with support for WE in bivariate analysis, and to limit multicollinearity with alternative socioeconomic measures (see Supporting Information Table S5 for variance inflation factor statistics, confirming moderate correlation between included independent variables). Women who made a cash income also remain significantly more likely to report that their husband is supportive even in the presence of related covariates. However, this association is absent in men's self-reported support for WE. Family size is not predictive of self-reported support for WE in the presence of additional covariates, while the relationship with polygyny remains, at least within the higher-powered model (Model 1), albeit at borderline statistical significance (i.e. *p* < 0.1, Table 2).

**Table 2.** Multivariate regression models predicting self- and wife-reported summary scores, and the discrepancy between scores

		**Self-reported summary score**		**Wife-reported summary score**	**Discrepancy score**
		**Model 1**	**Model 2**	**Model 3**	**Model 4**
		B [95% CI]	B [95% CI]	B [95% CI]	B [95% CI]
**Intercept**		59.84***	61.72***	48.13***	25.08**
		[53.06,66.62]	[50.78,72.66]	[32.95,63.32]	[9.81,40.35]
**Self-reported summary score** (mean centred)		—	—	—	0.84***
		—	—	—	[0.68,1.01]
**Education level** (reference: None)	Primary	9.59**	**8.19†**	9.44	−8.23
		[3.51,15.66]	[−1.55,17.93]	[−4.02,22.90]	[−21.71,5.24]
	Secondary	14.27***	12.15*	**12.52†**	−10.65
		[7.89,20.64]	[2.11,22.19]	[−1.36,26.40]	[−24.63,3.32]
	Higher	15.08***	10.31	8.78	−7.60
		[6.68,23.49]	[−2.88,23.49]	[−10.10,27.65]	[−26.45,11.24]
**Occupation** (reference: Subsistence)	Unskilled	2.30	2.02	2.68	−2.52
		[−1.42,6.01]	[−3.19,7.23]	[−4.76,10.12]	[−9.93,4.89]
	Skilled	**3.17†**	1.84	2.42	−2.11
		[−0.13,6.46]	[−2.74,6.42]	[−4.10,8.94]	[−8.62,4.39]
	Professional	8.00*	5.33	18.65*	−17.54*
		[1.86,14.13]	[−4.91,15.57]	[3.98,33.33]	[−32.21,−2.87]
**Subjective wealth rating** (reference: Poor/Very poor)	Average	1.31	2.14	0.84	−0.55
		[−1.08,3.71]	[−1.05,5.32]	[−3.70,5.37]	[−5.08,3.98]
	Wealthy	4.99**	7.24**	4.38	−3.39
		[1.48,8.51]	[2.39, 12.09]	[−2.59,11.34]	[−10.41,3.63]
**Wife's income** (reference: no income)	Earns Income	–	0.37	5.96**	−5.95**
		–	[−2.34,3.08]	[2.11,9.81]	[−9.78,−2.11]
**Number of children** (reference: 1–2)	3	0.83	0.66	1.58	−1.48
		[−1.93,3.60]	[−3.22,4.53]	[−3.88,7.04]	[−6.92,3.97]
	4	0.04	0.35	0.52	−0.35
		[−2.90,2.99]	[−3.70,4.40]	[−5.22,6.26]	[−6.07,5.37]
	5†	0.13	−0.07	−0.02	−0.02
		[−2.67,2.94]	[−3.96,3.81]	[−5.55,5.52]	[−5.53,5.49]
**Marriage** (reference: Monogamous)	Polygynous	**−3.57†**	−3.12	−1.99	1.55
		[−7.71,0.57]	[−8.73,2.50]	[−9.96,5.99]	[−6.41,9.51]
Model *N*		574	311	294	294
Model *R*^2^		0.14	0.10	0.11	0.28

*** *p* < 0.001; ** *p* < 0.01; * *p* < 0.05; † *p* < 0.1

### Which men are most likely to exaggerate support of women?

Men who claim the greatest support for WE seemingly exaggerate support for WE to the largest extent, as indicated by their greater discrepancy scores (Figure 3d). In regression models predicting discrepancy scores that adjust only for this relationship, relatively well-educated, professional and wealthy men, and those whose wife earns an income, have significantly lower discrepancy scores i.e. are less likely to report notably higher support of WE than estimated by their wives (Supporting Information Table S4). In full multivariate models, only a professional occupation and having a wife who earns a cash income remain independently predictive of relatively low discrepancies between self- and wife-reported estimates of support for WE (Table 2). Thus, with respect to occupation, professionals not only claim higher support for WE, but they are also more likely to agree with their wife's evaluation. In contrast, subsistence farmers reporter relatively low levels of support for WE and appear more likely to upwardly exaggerate the degree of their support. With respect to wife's income, ostensible differences in men's exaggeration tendencies mask higher support of WE among those whose wife earns a cash income in self-reported attitudes, which is only apparent in wife-reported measures (see also Figure 4, Table 2)

## Discussion

### Variation in men's support for women's empowerment

Men's support for WE appears limited within the study community, consistent with wider characterisations of Tanzania as having high gender inequality (Feinstein et al., 2010; Levtov et al., 2014; UNDP, 2018). Men were least supportive of WE when presented with statements implying a direct conflict of interest (e.g. authority in decision-making, acceptability of IPV). Domains that do not necessarily entail an explicit conflict or obvious cost to men garnered greater support. For example, a large majority of men ostensibly favour balanced sex ratios, women's labour market participation, participation in community meetings and girls’ education (see also Hedges et al. 2018 on emerging gender parity in schooling). This observation suggests that future studies would do well to disentangle which aspects of WE represent a true sexual conflict of interest, which will necessarily vary by time and place. This will require a dedicated theoretical and empirical consideration of the consequences of women's autonomy, rights and wellbeing for *both* genders/sexes rather than just women. Continued behavioural ecological studies of the human family, sensitive to vital importance of context, are well positioned to provide relevant data in this effort. Methodological refinement is also required to isolate and contrast domains of WE, which is only crudely estimated here. Indeed, our survey instrument is not ideal for quantitatively comparing support across different domains of WE, given differences in the phasing and the sensitivity of measurement across statements (Figure 2).

Domain comparisons aside, our composite measures of men's support for WE demonstrate internal consistency, indicating that a broad, encompassing concept of men's support of WE presents a valid construct for analysis. Well-educated, subjectively wealthier men, and those with high status occupations, were relatively more supportive of WE. Comparable relationships with education have been demonstrated in prior studies, indicating that such relationships are robust and generalisable (Charles, 2019; Kyoore & Sulemana, 2019; Levtov et al., 2014; Scott et al., 2014; Tran et al., 2016; Uthman et al., 2009). Positive impacts of high socioeconomic status may be accounted for by increased exposure to, and transmission of, extra-local and relatively egalitarian gender norms and/or relaxed gendered competition over family resources. Our analyses are not positioned to distinguish these alternatives. Men with professional occupations, for example, are more likely to be wealthier, but are also more likely to have experienced life outside of the town. However, men's income, an objective but incomplete measure of wealth, was not associated with support for WE (see also Levtov et al., 2014 for mixed relationships with income across different nations).

Consistent with positive effects of greater bargaining power within marriage, men whose wives earn an income were more supportive of WE. Causality, of course, may go in the opposite direction: men who are more accepting of women's economic independence may be more enabling of women's work. Future research could consider gendered divisions of labour associated with livelihood types, which have been predicted to influence patriarchal ideology in both anthropology (Draper, 1975; Hrdy, 1997; Smuts, 1995) and economics (Alesina et al., 2013; Becker, 2019; Hansen et al., 2015). In particular it would be insightful to leverage variation in livelihoods within mixed economies and those undergoing transition, rather than focus on population-level comparisons which may be vulnerable to confounding with alternative socioecological factors. Considering relative wealth between the genders, rather than just absolute wealth, may also be instructive, and help explain a lack of income effects. Increased women's relative economic status to their husbands, for example, has been argued to lead men to react negatively in attempt to retain the status quo via increased IPV (Abramsky et al., 2019; Cools & Kotsadam, 2017).

In contrast to socioeconomic status, demographic indicators were weak predictors of men's attitudes. Contrary to the prediction of Brooks and Blake (2019), we found no relationship between men's age and support for WE (see also inconsistent findings in Charles, 2019 and Levtov, 2014), although we note that the age range of men included in this study is not very wide. Also contrary to Brooks and Blake (2019), higher fertility was associated with slightly less, not more, support of WE, although this pattern disappeared once socioeconomic differences were considered. Polygynous men were less supportive of WE than monogamous men, consistent with idea that it is a marker of relative gender inequality, but this reached only borderline statistical significance, and only in men's self-reported attitudes. Finally, spousal age gap, another potential indicator of sexual conflict, was related to neither self- nor wife-reported attitudes. This is consistent with our findings that, although women frequently marry men older than their stated ideals in this population, the magnitude of spousal age gaps does not predict women's (self-reported) household decision-making authority, depressive symptomology or reproductive success among those married to older men (Lawson et al., 2021).

Our results do not exclude the possibility that demographic norms coevolve with support for WE at higher levels of aggregation (e.g. community-level). Multilevel analysis could explore this possibility, as has previously been utilised in studies of marriage type and wellbeing (Lawson et al., 2015; Smith-Greenaway & Trinitapoli, 2014). Smith-Greenaway and Trinitapoli (2014), for example, suggest that the degree to which polygyny is normative both reflects and influences women's status across a community a whole, in a way not effectively captured by contrasting currently monogamous and polygynous marriages within a population. Longitudinal analyses, comparing men before and after the birth of children or the addition of wives, could also better address causality. Baxter et al. (2015) using Australian panel data, for example, demonstrate that after the birth of a first child both men and women become more likely to support mothering as women's most important role in life. These results also confirm that individual gender ideology is flexible across the life course and responsive to key life events.

### Misrepresentation in men's support for women's empowerment

Prior studies of men's support for WE, reliant on direct survey techniques, have repeatedly acknowledged the possibility of social desirability bias (Charles, 2019; Kyoore & Sulemana, 2019; Lawoko, 2008; Levtov et al., 2014; Schuler et al., 2011; Vaillant et al., 2020). We provide a strong demonstration of that such concerns are warranted. Our results are consistent with men frequently exaggerating, often substantially, their support for WE, as indicated by wide discrepancies between self- and wife-reported measures. As might be expected, the most sensitive topics (e.g. attitudes to IPV) appear most prone to social desirability bias (Figure 2, Supporting Information Figure S2). There is also good reason to suspect that such patterns are generalisable to reports of recalled behaviour. The Tanzanian IMAGES survey (Levtov et al., 2018), for example, reports that men and women disagree on the frequency that sex is consensual and that IPV occurs, with men presenting their behaviour more favourably than women. While, Anderson et al. (2017), in a comprehensive analysis of husband and wife perceptions of decision-making authority across a large, nationally representative sample of Tanzanian farming households, report parallel results to the present study. Consistent with a tendency for men to exaggerate their support of WE, husbands report more authority in household decisions for their wives than wives report for themselves.

We also report evidence consistent with some men exaggerating more than others. This is evident from (a) the wide variance in discrepancy scores between self- and wife-reported measures, (b) the larger discrepancies between self- and wife-reported measures for men who claimed especially high support for WE and (c) observed socioeconomic variability. We hypothesised that high-status men would be most familiar with the socially ‘correct’ answer and so feel more compelled to exaggerate their views. The opposite appears true. While our results contradict our initial hypothesis, and the results of a prior study of men's attitudes to FGC (Gibson et al., 2018), more recent work (Gibson et al., 2020) also reports that less educated Ethiopian men were more likely to hide approval of IPV. Thus, social desirability bias may play out differently depending on the context. In this urbanising context, we speculate that all men are somewhat aware of the socially desirable answers. Instead a professional occupational status genuinely changes core attitudes, so that men's beliefs become more consistent with the interviewer – reducing the need to exaggerate. Men whose wife earns an income also appear less likely to exaggerate their support for WE, with potentially similar mechanisms at play. The presence of a foreign researcher during the interview was not impactful, perhaps because participants assumed that non-Tanzanian researchers could not understand Swahili.

Unexpectedly, a minority of men who report the very lowest support for WE tend to have their wife say he is *more supportive* than he claims himself (Figure 3d). Some participants found the survey entertaining, joking about the superiority of men between questions. Such men may therefore have been over-egging their lack of support for WE to perform masculine stereotypes. Another possibility is that these men misrepresented themselves to their wives who operate under a false impression that he is relatively supportive of WE. However, our survey topics are central to everyday life and thus we anticipate that women's estimates are based on direct experience of their husband's actual behaviour. Perhaps more feasibility, wives may feel shame admitting that the husband is especially unsupportive, or be concerned that reporting him as such carries a risk of being reprimanded. While we hope that this possibility was minimised by the use of in-private, same-gender interviews, this is a limitation to our study design, challenging our assumption that wife reports are necessarily relatively accurate estimates of men's true attitudes.

These considerations underline the difficulty of ever getting truly reliable estimates of attitudes on sensitive topics, or indeed recalled behaviours (see also Anderson et al., 2017), via survey methodologies. Considering the variable pathways to discrepant self- and wife-reported measures above, including our demonstration that discrepancies are related to both men's overall degree of support for WE and sociodemographic characteristics, it is unsurprising that self- and wife-reported measures of men's support for WE were only weakly correlated (Figure 3b). This result is immediately concerning, implying that past and future studies of men's self-reported support for WE may only be crudely indicative of true underlying attitudes. Paralleling our findings, Anderson et al. (2017) also report considerable variation in the degree of accord between husband and wife reports of household decision-making. However, more reassuringly, with some exceptions (e.g. monogamous vs polygynous marriage, whether or not the wife earns an income, Figure 4) general trends with sociodemographic variables are broadly similar across self- and wife-reported measures of support for WE.

Distinguishing potential sources of misrepresentation in self-reported attitudes presents a fundament challenge to better understanding the measurement and continuing evolution of gender norms and ideology. Without rising to this challenge, we are left with the unsatisfactory conclusion that estimated support for WE fundamentally hinges on who is surveyed. We also speculate that issues of false representation mean that men (and women) themselves may face difficulty in accurately assessing local social norms, with potential consequences for norm change. Bursztyn et al. (2020), for example, recently reported that men in Saudi Arabia tend to believe that other men are less supportive of WE than they are themselves (a finding that appears somewhat generalisable, e.g. see also Sobotka, 2020), and, furthermore, that correcting such misconceptions promotes positive change in men's behaviour. Thus, men appear keen to conform to local norms, but have difficulty judging them, perhaps because of widespread misrepresentation of individual beliefs. There is clear scope for scholars of cultural evolution (Creanza et al., 2017; Mesoudi, 2011) to address such questions of norm perception in future research, and in doing so more actively contribute to global health research and intervention design.

## Conclusion

Global health research on WE increasingly adopts a social norms framework, wherein patriarchal ideology represents a fundamental barrier to gender equality (Jayachandran, 2020; UNDP, 2020). In contrast, our results add to a growing quantitative (Levtov et al., 2014) and qualitative literature (Dworkin et al., 2013; Pierotti et al., 2018; Wyrod, 2008) documenting considerable diversity in men's attitudes to WE within populations. Focusing on variability offers a distinct starting point for initiatives that might otherwise conceptualise all men as equally unsupportive of WE. For example, the success of initiatives may be boosted by recruiting already supportive men into intervention design as role models and social influencers. Alternatively, understanding variability may enable interventions to target men for whom change would be most transformative. It is notable, for instance, that according to wives’ estimates men in (currently) polygynous as opposed to monogamous marriages and men married to especially young wives were not less supportive of WE, challenging common assumptions about the costs of such marriage forms for women. Interventions may therefore do better to target men on the basis of alternative factors, such as lower educational attainment and/or when wives do not work outside of the home.

Our findings also strongly support further development of indirect survey techniques (see also Gibson et al., 2018, 2020; Lindstrom et al., 2010; Nillesen et al., 2021), reinforcing concerns about the validity of previously demonstrated sociodemographic variation in men's attitudes based only on self-report data. We have argued that a tendency to frequently, and often substantially, exaggerate support for WE under direct questioning best explains observed differences between self- and wife-reported measures. We acknowledge that this conclusion is undermined if wife-reported measures are also strongly influenced by social desirability bias and/or women are ill-informed of their husbands’ views. Ultimately, no method is perfect and the assumptions of our approach should not go without scrutiny. Until the invention of literal mindreaders, diversifying our toolkit offers our best route forward to quantifying and ultimately transforming men's attitudes and support for WE worldwide. We are optimistic that continued collaboration and dialogue between global health scholars and evolutionary human scientists, bringing new tools and ideas to the table (see also Gibson & Lawson, 2015), can create novel pathways to innovation and progress towards our shared goals.

## Supplementary material

For supplementary material accompanying this paper visit https://doi.org/10.1017/ehs.2021.23. click here to view supplementary material

## Research transparency and reproducibility

The data that support the findings in this study are available from the corresponding author and the National Institute for Medical Research, Tanzania, on request, providing that necessary steps are taken to ensure participant anonymity is maintained. Raw data is not suitable for being made publicly available owing to its sensitive nature and the possibility of participant identification.
