# Supplementary material for: He for she? Variation and exaggeration in men's support for women's empowerment in northern Tanzania
Source: Evol Hum Sci. 2021 Mar 18;3:e27. doi: 10.1017/ehs.2021.23 (PMC10427278; doi:10.1017/ehs.2021.23)
Supplement: Supplementary file 1 [file S2513843X21000232sup001.docx]

Supplementary Information for ‘***He for She? Variation and Exaggeration in Men’s Support for Women’s Empowerment in Northern Tanzania’***

David W. Lawson, Susan B. Schaffnit, Joseph A. Kilgallen, Yusufu Kumogola, Anthony Galura and Mark Urassa.

| **Table S1: Table showing Swahili translations of survey questions.** | |
| --- | --- |
| Statement in English | Statement in Swahili |
| A. A man should have the final say about decisions in his home | Mwanaume ndiye mwenye mamlaka ya kutoa maamuzi nyumbani |
| B. A woman should be free to divorce (or leave) her husband even if he does not wish | Mwanamke ana uhuru wa kuachana (kumtaliki) na mmewe hata kama mme hataki ndoa iishe |
| C. A wife should be able to prevent her husband from taking another wife | Mke anaweza kumzuia mme wake kuoa mwanamke mwingine, endapo mme atakuwa tofauti na matakwa yake |
| D. A man is the one who decides when to have sex with his wife | Mwanaume ndiye huwa anaamua ni wakati gani wa kujamiana na mkewe |
| E. A husband and wife should decide together equally about when to have children. | Mme na mke wanapaswa kuamua kwa pamoja, ni wakati gani wa kupata watoto |
| F. A man is justified in hitting his wife if she argues with him | Mwanaume ana haki ya kumpiga mkewe endapo atabishana naye |
| G. A man is justified in hitting his wife if she refuses to have sex with him | Mwanaume ana haki ya kumpiga mkewe endapo atakataa kujamiana naye |
| H. A woman should tolerate being beaten by her husband to keep her family together | Mwanamke anatakiwa kuvumilia kipigo kutoka kwa mmewe ili waitunze familia yao kwa pamoja |
| I. It is the mother’s responsibility alone to take care of the children | Ni jukumu la mama peke yake kutunza/kulea watoto |
| J. If a woman wants to avoid being pregnant, it is her responsibility alone to prevent the pregnancy | Kama mwanamke anataka kujizuia asipate ujauzito, ni jukumu lake peke yake kujizuia asipate ujauzito |
| K. Only men should be allowed to own land | Ni wanaume peke yao ndio wanapaswa kumiliki ardhi |
| L. Only men should be allowed to manage a business | Ni wanaume peke yao ndio wanapaswa kufanya biashara. |
| M. It is important for women to earn their own money | Ni muhimu kwa wanawake kupata fedha zao wenyewe. |
| N. Women should be welcome at community meetings | Wanawake wanapaswa kukaribishwa kufika kwenye mikutano ya kjamii |
| O. Women should express their opinions at community meetings | Wanawake wanapaswa kutoa ushauri wao kwenye mikutano ya kjamii |
| P. It is better to have more sons than daughters in a family | Ni vizuri zaidi kuwa na watoto wa kiume kuliko wa kike katika familia |
| Q. Education is more important for boys than girls | Elimu ni muhimu zaidi kwa wavulana kuliko wasichana |
| R. It is important for girls/women to be educated | Ni muhimu kwa wasichana/wanawake kupata elimu |
| S. A woman can live a successful life even if she does not marry a man | Mwanamke anaweza kuishi maisha mazuri hata kama hajaolewa na mwanaume |
| T. Only when a woman has a child is she a real woman | Mwanamke anakuwa mwanamke kamili wakati anapokuwa na mtoto tu |

| **Table S2: Characteristics of Unpaired and Paired Men** | | | | |
| --- | --- | --- | --- | --- |
| Variable | | Unpaired  (n = 273) | Paired  (n = 317) | Test Statistic** |
| **Age (Years)** | Mean (SD) | 34.47 (4.88) | 34.81 (4.65) | t = -0.8,  df = 565.79,  p = 0.381 |
|  | Range | 23.00 - 45.00 | 24.00 - 45.00 |  |
| **Man’s Highest Education Level Achieved** | Missing | 1 | 0 | x2 = 4.49, df = 3  p = 0.213 |
|  | No education | 11 (4.0%) | 6 (1.9%) |  |
|  | Primary school | 177 (65.1%) | 220 (69.4%) |  |
|  | Secondary school/technical training | 65 (23.9%) | 77 (24.3%) |  |
|  | Higher education | 19 (7.0%) | 14 (4.4%) |  |
| **Subjective Wealth Rating** | Wealthy | 44 (16.1%) | 45 (14.2%) | x2 = 0.89, df = 2  p = 0.642 |
|  | Average | 144 (52.7%) | 179 (56.5%) |  |
|  | Poor | 85 (31.1%) | 93 (29.3%) |  |
| **Income past week (Tanzanian Shillings)** | Missing | 2 | 2 | x2 = 3.56, df = 3  p = 0.313 |
|  | No income | 51 (18.8%) | 48 (15.2%) |  |
|  | Low income | 66 (24.4%) | 83 (26.3%) |  |
|  | Medium income | 73 (26.9%) | 102 (32.4%) |  |
|  | High income | 81 (29.9%) | 82 (26.0%) |  |
| **Occupation** | N-Miss | 9 | 5 | x2 = 8.07, df = 3  **p = 0.045** |
|  | Subsistence | 31 (11.7%) | 30 (9.6%) |  |
|  | Unskilled | 57 (21.6%) | 61 (19.6%) |  |
|  | Skilled | 151 (57.2%) | 207 (66.3%) |  |
|  | Professional | 25 (9.5%) | 14 (4.5%) |  |
| **Number of Children** | 1-2 | 97 (35.5%) | 79 (24.9%) | x2 = 9.35, df = 3  **p = 0.025** |
|  | 3 | 62 (22.7%) | 75 (23.7%) |  |
|  | 4 | 49 (17.9%) | 61 (19.2%) |  |
|  | 5+ | 65 (23.8%) | 102 (32.2%) |  |
| **Number of Wives** | Monogamous | 257 (94.1%) | 297 (93.7%) | x2 = 0.00, df = 1  p = 0.957 |
|  | Polygynous | 16 (5.9%) | 20 (6.3%) |  |
| **Mzungu present?** | No mzungu present | 221 (81.0%) | 222 (70.0%) | x2 = 8.78, df = 1  **p = 0.003** |
|  | Mzungu present | 52 (19.0%) | 95 (30.0%) |  |
| * Unpaired = Men whose wives were not sampled; Paired = Men whose wives were also sampled.  ** t-test for age, chi-squared test for all others. | | | | |

| **Table S3 Self and Wife-Reported Views on Women’s Empowerment** | | | | |
| --- | --- | --- | --- | --- |
| **Statement** | **Response** | **Self-Report** | **Wife-Report** | **Wilcoxon rank-sum test*** |
| A. A man should have the final say about decisions in his home | Strongly agree | 234 (39.7%) | 178 (56.2%) | W = 73885, **p<0.001** |
|  | Agree | 136 (23.1%) | 45 (14.2%) |  |
|  | Neutral | 13 (2.2%) | 4 (1.3%) |  |
|  | Disagree | 96 (16.3%) | 41 (12.9%) |  |
|  | Strongly disagree | 110 (18.6%) | 38 (12.0%) |  |
|  | Don’t know | 0 (0.0%) | 8 (2.5%) |  |
|  | Refusal | 1 (0.2%) | 3 (0.9%) |  |
| B. A woman should be free to divorce (or leave) her husband even if he does not wish | Strongly agree | 107 (18.1%) | 49 (15.5%) | W = 95861, **p<0.001** |
|  | Agree | 180 (30.5%) | 39 (12.3%) |  |
|  | Neutral | 37 (6.3%) | 13 (4.1%) |  |
|  | Disagree | 114 (19.3%) | 69 (21.8%) |  |
|  | Strongly disagree | 148 (25.1%) | 108 (34.1%) |  |
|  | Don’t know | 2 (0.3%) | 35 (11.0%) |  |
|  | Refusal | 2 (0.3%) | 4 (1.3%) |  |
| C. A wife should be able to prevent her husband from taking another wife | Strongly agree | 126 (21.4%) | 25 (7.9%) | W = 110384, **p<0.001** |
|  | Agree | 180 (30.5%) | 43 (13.6%) |  |
|  | Neutral | 23 (3.9%) | 21 (6.6%) |  |
|  | Disagree | 138 (23.4%) | 61 (19.2%) |  |
|  | Strongly disagree | 121 (20.5%) | 130 (41.0%) |  |
|  | Don’t know | 0 (0.0%) | 33 (10.4%) |  |
|  | Refusal | 2 (0.3%) | 4 (1.3%) |  |
| D. A man is the one who decides when to have sex with his wife | Strongly agree | 95 (16.1%) | 113 (35.6%) | W = 64823, **p<0.001** |
|  | Agree | 80 (13.6%) | 54 (17.0%) |  |
|  | Neutral | 43 (7.3%) | 6 (1.9%) |  |
|  | Disagree | 182 (30.8%) | 58 (18.3%) |  |
|  | Strongly disagree | 187 (31.7%) | 67 (21.1%) |  |
|  | Don’t know | 1 (0.2%) | 16 (5.0%) |  |
|  | Refusal | 2 (0.3%) | 3 (0.9%) |  |
| E. A husband and wife should decide together equally about when to have children. | Strongly agree | 382 (64.7%) | 206 (65.0%) | W = 81090, p = 0.1804 |
|  | Agree | 170 (28.8%) | 65 (20.5%) |  |
|  | Neutral | 9 (1.5%) | 3 (0.9%) |  |
|  | Disagree | 16 (2.7%) | 13 (4.1%) |  |
|  | Strongly disagree | 7 (1.2%) | 4 (1.3%) |  |
|  | Don’t know | 4 (0.7%) | 22 (6.9%) |  |
|  | Refusal | 2 (0.3%) | 4 (1.3%) |  |
| F. A man is justified in hitting his wife if she argues with him | Strongly agree | 51 (8.6%) | 111 (35.0%) | W = 52126, **p<0.001** |
|  | Agree | 100 (16.9%) | 70 (22.1%) |  |
|  | Neutral | 21 (3.6%) | 13 (4.1%) |  |
|  | Disagree | 186 (31.5%) | 46 (14.5%) |  |
|  | Strongly disagree | 231 (39.2%) | 59 (18.6%) |  |
|  | Don’t know | 0 (0.0%) | 14 (4.4%) |  |
|  | Refusal | 1 (0.2%) | 4 (1.3%) |  |
| G. A man is justified in hitting his wife if she refuses to have sex with him | Strongly agree | 15 (2.5%) | 74 (23.3%) | W = 53625, **p<0.001** |
|  | Agree | 17 (2.9%) | 30 (9.5%) |  |
|  | Neutral | 7 (1.2%) | 6 (1.9%) |  |
|  | Disagree | 145 (24.6%) | 80 (25.2%) |  |
|  | Strongly disagree | 404 (68.5%) | 112 (35.3%) |  |
|  | Don’t know | 0 (0.0%) | 12 (3.8%) |  |
|  | Refusal | 2 (0.3%) | 3 (0.9%) |  |
| H. A woman should tolerate being beaten by her husband to keep her family together | Strongly agree | 62 (10.5%) | 79 (24.9%) | W = 68631, **p<0.001** |
|  | Agree | 108 (18.3%) | 43 (13.6%) |  |
|  | Neutral | 14 (2.4%) | 10 (3.2%) |  |
|  | Disagree | 133 (22.5%) | 60 (18.9%) |  |
|  | Strongly disagree | 272 (46.1%) | 98 (30.9%) |  |
|  | Don’t know | 0 (0.0%) | 23 (7.3%) |  |
|  | Refusal | 1 (0.2%) | 4 (1.3%) |  |
| I. It is the mother’s responsibility alone to take care of the children | Strongly agree | 42 (7.1%) | 47 (14.8%) | W = 77886, **p<0.001** |
|  | Agree | 26 (4.4%) | 20 (6.3%) |  |
|  | Neutral | 4 (0.7%) | 7 (2.2%) |  |
|  | Disagree | 158 (26.8%) | 74 (23.3%) |  |
|  | Strongly disagree | 357 (60.5%) | 157 (49.5%) |  |
|  | Don’t know | 0 (0.0%) | 9 (2.8%) |  |
|  | Refusal | 3 (0.5%) | 3 (0.9%) |  |
| J. If a woman wants to avoid being pregnant, it is her responsibility alone to prevent the pregnancy | Strongly agree | 48 (8.1%) | 62 (19.6%) | W = 67274, **p<0.001** |
|  | Agree | 39 (6.6%) | 38 (12.0%) |  |
|  | Neutral | 7 (1.2%) | 7 (2.2%) |  |
|  | Disagree | 204 (34.6%) | 82 (25.9%) |  |
|  | Strongly disagree | 290 (49.2%) | 105 (33.1%) |  |
|  | Don’t know | 1 (0.2%) | 19 (6.0%) |  |
|  | Refusal | 1 (0.2%) | 4 (1.3%) |  |
| K. Only men should be allowed to own land | Strongly agree | 23 (3.9%) | 43 (13.6%) | W = 75646, **p<0.001** |
|  | Agree | 33 (5.6%) | 21 (6.6%) |  |
|  | Neutral | 7 (1.2%) | 2 (0.6%) |  |
|  | Disagree | 189 (32.0%) | 84 (26.5%) |  |
|  | Strongly disagree | 337 (57.1%) | 145 (45.7%) |  |
|  | Don’t know | 0 (0.0%) | 18 (5.7%) |  |
|  | Refusal | 1 (0.2%) | 4 (1.3%) |  |
| L. Only men should be allowed to manage a business | Strongly agree | 17 (2.9%) | 29 (9.1%) | W = 84645, p = 0.1259 |
|  | Agree | 25 (4.2%) | 18 (5.7%) |  |
|  | Neutral | 7 (1.2%) | 3 (0.9%) |  |
|  | Disagree | 185 (31.4%) | 77 (24.3%) |  |
|  | Strongly disagree | 355 (60.2%) | 177 (55.8%) |  |
|  | Don’t know | 0 (0.0%) | 9 (2.8%) |  |
|  | Refusal | 1 (0.2%) | 4 (1.3%) |  |
| M. It is important for women to earn their own money | Strongly agree | 229 (38.8%) | 192 (60.6%) | W = 69522, **p<0.001** |
|  | Agree | 230 (39.0%) | 61 (19.2%) |  |
|  | Neutral | 16 (2.7%) | 4 (1.3%) |  |
|  | Disagree | 57 (9.7%) | 19 (6.0%) |  |
|  | Strongly disagree | 56 (9.5%) | 27 (8.5%) |  |
|  | Don’t know | 0 (0.0%) | 10 (3.2%) |  |
|  | Refusal | 2 (0.3%) | 4 (1.3%) |  |
| N. Women should be welcome at community meetings | Strongly agree | 380 (64.4%) | 228 (71.9%) | W = 80631, **p<0.001** |
|  | Agree | 188 (31.9%) | 64 (20.2%) |  |
|  | Neutral | 2 (0.3%) | 0 (0.0%) |  |
|  | Disagree | 13 (2.2%) | 6 (1.9%) |  |
|  | Strongly disagree | 6 (1.0%) | 6 (1.9%) |  |
|  | Don’t know | 0 (0.0%) | 9 (2.8%) |  |
|  | Refusal | 1 (0.2%) | 4 (1.3%) |  |
| O. Women should express their opinions at community meetings | Strongly agree | 403 (68.3%) | 229 (72.2%) | W = 84416, p = 0.06246 |
|  | Agree | 172 (29.2%) | 64 (20.2%) |  |
|  | Neutral | 1 (0.2%) | 2 (0.6%) |  |
|  | Disagree | 8 (1.4%) | 6 (1.9%) |  |
|  | Strongly disagree | 5 (0.8%) | 4 (1.3%) |  |
|  | Don’t know | 0 (0.0%) | 8 (2.5%) |  |
|  | Refusal | 1 (0.2%) | 4 (1.3%) |  |
| P. It is better to have more sons than daughters in a family | Strongly agree | 22 (3.7%) | 37 (11.7%) | W = 81550, p = 0.08431 |
|  | Agree | 26 (4.4%) | 14 (4.4%) |  |
|  | Neutral | 14 (2.4%) | 5 (1.6%) |  |
|  | Disagree | 182 (30.8%) | 74 (23.3%) |  |
|  | Strongly disagree | 344 (58.3%) | 166 (52.4%) |  |
|  | Don’t know | 1 (0.2%) | 17 (5.4%) |  |
|  | Refusal | 1 (0.2%) | 4 (1.3%) |  |
| Q. Education is more important for boys than girls | Strongly agree | 29 (4.9%) | 41 (12.9%) | W = 74197, **p<0.001** |
|  | Agree | 30 (5.1%) | 25 (7.9%) |  |
|  | Neutral | 5 (0.8%) | 3 (0.9%) |  |
|  | Disagree | 150 (25.4%) | 76 (24.0%) |  |
|  | Strongly disagree | 374 (63.4%) | 154 (48.6%) |  |
|  | Don’t know | 1 (0.2%) | 14 (4.4%) |  |
|  | Refusal | 1 (0.2%) | 4 (1.3%) |  |
| R. It is important for girls/women to be educated | Strongly agree | 482 (81.7%) | 236 (74.4%) | W = 92488, p = 0.1548 |
|  | Agree | 104 (17.6%) | 59 (18.6%) |  |
|  | Neutral | 0 (0.0%) | 1 (0.3%) |  |
|  | Disagree | 1 (0.2%) | 5 (1.6%) |  |
|  | Strongly disagree | 2 (0.3%) | 1 (0.3%) |  |
|  | Don’t know | 0 (0.0%) | 11 (3.5%) |  |
|  | Refusal | 1 (0.2%) | 4 (1.3%) |  |
| S. A woman can live a successful life even if she does not marry a man | Strongly agree | 257 (43.6%) | 147 (46.4%) | W = 82254, p = 0.4077 |
|  | Agree | 207 (35.1%) | 77 (24.3%) |  |
|  | Neutral | 22 (3.7%) | 4 (1.3%) |  |
|  | Disagree | 54 (9.2%) | 24 (7.6%) |  |
|  | Strongly disagree | 46 (7.8%) | 38 (12.0%) |  |
|  | Don’t know | 2 (0.3%) | 23 (7.3%) |  |
|  | Refusal | 2 (0.3%) | 4 (1.3%) |  |
| T. Only when a woman has a child is she a real woman | Strongly agree | 83 (14.1%) | 102 (32.2%) | W = 59000, **p<0.001** |
|  | Agree | 66 (11.2%) | 47 (14.8%) |  |
|  | Neutral | 9 (1.5%) | 12 (3.8%) |  |
|  | Disagree | 185 (31.4%) | 52 (16.4%) |  |
|  | Strongly disagree | 245 (41.5%) | 74 (23.3%) |  |
|  | Don’t know | 1 (0.2%) | 26 (8.2%) |  |
|  | Refusal | 1 (0.2%) | 4 (1.3%) |  |
| Wilcoxon rank-sum tests exclude ‘Don’t know’ and ‘Refusal’ response categories. | | | | |

| **Table S4: Regression Models Predicting Self and Wife-Reported Summary Scores, and the Discrepancy Between Scores** | | | | | |
| --- | --- | --- | --- | --- | --- |
|  | | Self-Reported Summary Score | Wife-Reported Summary Score | Discrepancy Score | |
|  | | Bivariate | Bivariate | Bivariate | Adjusted for Self-Report Summary Score |
|  |  | B [95%CI) | B [95%CI) | B [95%CI) | B [95%CI) |
| Age  (reference: <30) | 30-34 | 2.04 | 1.68 | 1.74 | -0.86 |
|  |  | [-1.08, 5.16] | [-4.18, 7.54] | [-4.81, 8.29] | [-6.66, 4.95] |
|  | 35-39 | 2.10 | 1.91 | -0.41 | -1.55 |
|  |  | [-0.93, 5.12] | [-3.93, 7.75] | [-6.94, 6.12] | [-7.32, 4.22] |
|  | 40+ | 1.45 | 3.77 | -4.36 | -3.92 |
|  |  | [-2.05, 4.94] | [-2.84, 10.39] | [-11.76, 3.04] | [-10.45, 2.62] |
| Education Level  (reference: None) | Primary | **10.38 ***** | 8.51 | 0.30 | -6.94 |
|  |  | [4.30, 16.46] | [-4.82, 21.84] | [-14.96, 15.56] | [-20.27, 6.39] |
|  | Secondary | **16.33 ***** | **12.52 +** | 1.31 | -10.06 |
|  |  | [10.04, 22.62] | [-1.14, 26.18] | [-14.33, 16.95] | [-23.83, 3.71] |
|  | Higher | **22.65 ***** | **22.29 **** | -5.85 | **-19.36 *** |
|  |  | [15.39, 29.92] | [6.41, 38.18] | [-24.04, 12.33] | [-35.38, -3.35] |
| Occupation  (reference: Subsistence) | Unskilled | 1.55 | 2.38 | -2.58 | -2.42 |
|  |  | [-2.23, 5.34] | [-4.98, 9.74] | [-10.93, 5.77] | [-9.72, 4.88] |
|  | Skilled | **4.63 **** | 3.83 | -0.91 | -3.23 |
|  |  | [1.31, 7.95] | [-2.63, 10.29] | [-8.25, 6.42] | [-9.66, 3.19] |
|  | Professional | **15.04 ***** | **20.57 ***** | -9.50 | **-18.30 ***** |
|  |  | [10.12, 19.96] | [10.10, 31.04] | [-21.37, 2.38] | [-28.83, -7.76] |
| Subjective Wealth Rating  (reference: poor/v poor) | Average | **3.36 **** | 2.37 | 0.46 | -1.85 |
|  |  | [1.08, 5.64] | [-1.89, 6.63] | [-4.40, 5.33] | [-6.10, 2.41] |
|  | Wealthy | **9.80 ***** | **9.77 **** | -1.10 | **-8.17 **** |
|  |  | [6.63, 12.96] | [3.75, 15.78] | [-7.97, 5.77] | [-14.31, -2.03] |
| Income Level  (reference: No income) | Low | -2.19 | **-9.10 **** | **6.10 +** | **8.52 **** |
|  |  | [-5.41, 1.03] | [-15.22, -2.99] | [-0.89, 13.09] | [2.43, 14.60] |
|  | Medium | 0.37 | -4.46 | 3.99 | 4.37 |
|  |  | [-2.74, 3.49] | [-10.32, 1.39] | [-2.70, 10.68] | [-1.43, 10.18] |
|  | High | **3.50 *** | -1.13 | 3.04 | 1.51 |
|  |  | [0.35, 6.66] | [-7.16, 4.90] | [-3.85, 9.93] | [-4.48, 7.49] |
| Wife’s Income  (reference: No Income) | Earns Income | 0.88 | **5.42 **** | **-4.58 *** | **-5.23 **** |
|  |  | [-1.88, 3.64] | [1.59, 9.25] | [-8.91, -0.25] | [-9.02, -1.44] |
| Number of Children  (reference: 1-2) | 3 | -0.92 | -0.37 | -0.73 | 0.12 |
|  |  | [-3.77, 1.93] | [-5.78, 5.04] | [-6.83, 5.36] | [-5.23, 5.48] |
|  | 4 | -1.69 | -0.31 | 0.37 | 0.32 |
|  |  | [-4.73, 1.35] | [-6.06, 5.44] | [-6.11, 6.85] | [-5.37, 6.01] |
|  | 5+ | **-3.16 *** | -2.84 | -0.13 | 2.17 |
|  |  | [-5.86, -0.45] | [-7.94, 2.26] | [-5.87, 5.61] | [-2.89, 7.24] |
| Spousal Age Gap (reference: Wife is 1-4 years younger) | Same age/older | 0.22 | -3.63 | 3.44 | 3.59 |
|  |  | [-4.35, 4.79] | [-10.24, 2.98] | [-3.99, 10.88] | [-2.94, 10.12] |
|  | 5-8 years | 0.22 | -2.07 | 2.85 | 2.25 |
|  |  | [-3.10, 3.54] | [-6.72, 2.58] | [-2.38, 8.09] | [-2.35, 6.85] |
|  | 9+ years | 1.26 | 0.46 | 1.31 | -0.05 |
|  |  | [-2.60, 5.11] | [-4.92, 5.83] | [-4.73, 7.35] | [-5.36, 5.27] |
| Marital Status  (reference: Monogamous) | Poly | **-4.34 *** | -2.16 | -0.21 | 1.61 |
|  |  | [-8.64, -0.04] | [-9.93, 5.61] | [-8.94, 8.52] | [-6.08, 9.30] |
| Mzungu present  (reference: No) | Yes | 0.11 | 0.24 | 1.73 | 1.12 |
|  |  | [-2.28, 2.50] | [-5.81, 6.29] | [-2.94, 6.39] | [-2.99, 5.23] |
| Model N | | 316-589 | 295-299 | 295-299 | 295-299 |
| Model R2 | | 0.00 – 0.10 | 0.00 – 0.05 | 0.00 – 0.01 | 0.23-0.26 |

| **Table S5: Multivariate Regression Models Predicting Self and Wife-Reported Summary Scores, and the Discrepancy Between Scores**  **(Variance Inflation Factor Included)** | | | | | | | | | |
| --- | --- | --- | --- | --- | --- | --- | --- | --- | --- |
|  | | Self-Reported Summary Score | | | | Wife-Reported Summary Score | | Discrepancy Score | |
|  |  | Model 1 | | Model 2 | | Model 3 | | Model 4 | |
|  |  | B [95%CI) | VIF | B [95%CI) | VIF | B [95%CI) | VIF | B [95%CI) | VIF |
| Intercept | | **59.84 ***** | - | **61.72 ***** | **-** | **48.13 ***** | - | **25.08 **** | - |
|  |  | [53.06, 66.62] |  | [50.78, 72.66] |  | [32.95, 63.32] |  | [9.81, 40.35] |  |
| Self-Reported Summary Score  (mean centered) | | - | - | - | - | - | - | **0.84 ***** | 1.12 |
|  |  |  |  |  |  |  |  | [0.68, 1.01] |  |
| Education Level  (reference: None) | Primary | **9.59 **** | 2.22 | **8.19 +** | 2.41 | 9.44 | 2.63 | -8.23 | 2.73 |
|  |  | [3.51, 15.66] |  | [-1.55, 17.93] |  | [-4.02, 22.90] |  | [-21.71, 5.24] |  |
|  | Secondary | **14.27 ***** |  | **12.15 *** |  | **12.52 +** |  | -10.65 |  |
|  |  | [7.89, 20.64] |  | [2.11, 22.19] |  | [-1.36, 26.40] |  | [-24.63, 3.32] |  |
|  | Higher | **15.08 ***** |  | 10.31 |  | 8.78 |  | -7.60 |  |
|  |  | [6.68, 23.49] |  | [-2.88, 23.49] |  | [-10.10, 27.65] |  | [-26.45, 11.24] |  |
| Occupation  (reference: Subsistence) | Unskilled | 2.30 | 2.29 | 2.02 | 2.64 | 2.68 | 2.87 | -2.52 | 2.89 |
|  |  | [-1.42, 6.01] |  | [-3.19, 7.23] |  | [-4.76, 10.12] |  | [-9.93, 4.89] |  |
|  | Skilled | **3.17 +** |  | 1.84 |  | 2.42 |  | -2.11 |  |
|  |  | [-0.13, 6.46] |  | [-2.74, 6.42] |  | [-4.10, 8.94] |  | [-8.62, 4.39] |  |
|  | Professional | **8.00 *** |  | 5.33 |  | **18.65 *** |  | **-17.54 *** |  |
|  |  | [1.86, 14.13] |  | [-4.91, 15.57] |  | [3.98, 33.33] |  | [-32.21, -2.87] |  |
| Subjective Wealth Rating  (reference: poor/v poor) | Average | 1.31 | 1.43 | 2.14 | 1.50 | 0.84 | 1.55 | -0.55 | 1.58 |
|  |  | [-1.08, 3.71] |  | [-1.05, 5.32] |  | [-3.70, 5.37] |  | [-5.08, 3.98] |  |
|  | Wealthy | **4.99 **** |  | **7.24 **** |  | 4.38 |  | -3.39 |  |
|  |  | [1.48, 8.51] |  | [2.39, 12.09] |  | [-2.59, 11.34] |  | [-10.41, 3.63] |  |
| Wife’s Income  (reference: No Income) | Earns Income | – | - | 0.37 | 1.03 | **5.96 **** | 1.04 | **-5.95 **** | 1.04 |
|  |  |  |  | [-2.34, 3.08] |  | [2.11, 9.81] |  | [-9.78, -2.11] |  |
| Number of Children  (reference: 1-2) | 3 | 0.83 | 1.24 | 0.66 | 1.33 | 1.58 | 1.33 | -1.48 | 1.33 |
|  |  | [-1.93, 3.60] |  | [-3.22, 4.53] |  | [-3.88, 7.04] |  | [-6.92, 3.97] |  |
|  | 4 | 0.04 |  | 0.35 |  | 0.52 |  | -0.35 |  |
|  |  | [-2.90, 2.99] |  | [-3.70, 4.40] |  | [-5.22, 6.26] |  | [-6.07, 5.37] |  |
|  | 5+ | 0.13 |  | -0.07 |  | -0.02 |  | -0.02 |  |
|  |  | [-2.67, 2.94] |  | [-3.96, 3.81] |  | [-5.55, 5.52] |  | [-5.53, 5.49] |  |
| Marriage (reference: Monogamous) | Polygynous | **-3.57 +** | 1.07 | -3.12 | 1.12 | -1.99 | 1.13 | 1.55 | 1.13 |
|  |  | [-7.71, 0.57] |  | [-8.73, 2.50] |  | [-9.96, 5.99] |  | [-6.41, 9.51] |  |
| Model N | | 574 | | 311 | | 294 | | 294 | |
| Model R2 | | 0.14 | | 0.10 | | 0.11 | | 0.28 | |
| *** p < 0.001; ** p < 0.01; * p < 0.05; + p < 0.1 | | | | | | | | | |

**Figure S1: Self-reported support for women’s empowerment for full sample and paired men only.**

Figure shows sum percentage which strongly disagreed or disagree on left side, and sum percentage which agree or strongly agreed on right side for each statement. Maximum sample sizes are 590 for self-reported full sample and 317 self-reported were paired wife data is also available.


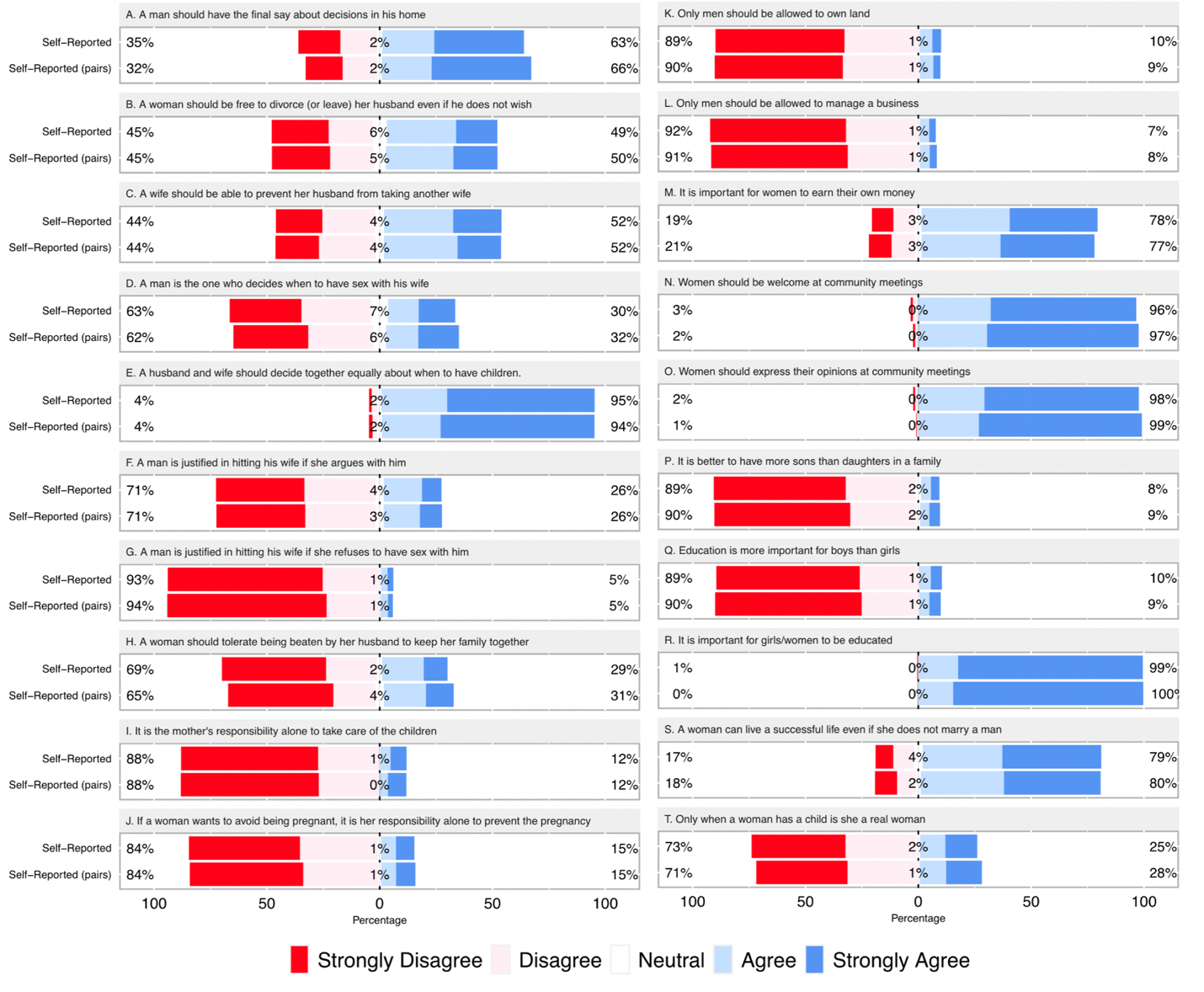


**Figure S2: Histograms showing discrepancy score between self and wife-reported attitudes by item.**

See Table 1 to match statements to the letter key. The level of discrepancy was calculated by first coding individual responses 1-5 for all statements from strongly disagree to strongly agree respectively where greater agreement is more supportive of WE, and reverse coding scores where the opposite is true, and then subtracting the self-report score from the wife-support score. While useful in identifying the source of overall discrepancies in the level of support for WE between self and wife-reported summary scores, we note that, due differences in the phrasing of statements (and so precision in measuring latent constructs of interest), the comparative magnitude of discrepancies should be interpreted with caution. A score of 0 indicates that husband and wives gave the same response for a particular statement. A positive value indicates that husbands report greater support for women’s empowerment than their wife estimates. This analysis confirms notable variation in discrepancy levels between self and wife-rated attitudes across most measures, and considerable variability in the degree of discrepancy within measures (see main text). In other words, some statements are characterized by larger spousal discrepancy than others, and there is much variability in the magnitude of discrepancies within statements across husband-wife pairs.
